# Chronic Exposure to Normobaric Hypoxia Increases Testosterone Levels and Testosterone/Cortisol Ratio in Cyclists

**DOI:** 10.3390/ijerph19095246

**Published:** 2022-04-26

**Authors:** Miłosz Czuba, Kamila Płoszczyca, Katarzyna Kaczmarczyk, Józef Langfort, Robert Gajda

**Affiliations:** 1 Faculty of Rehabilitation, Józef Piłsudski University of Physical Education in Warsaw, 00-968 Warsaw, Poland; milosz.czuba@awf.edu.pl (M.C.); katarzyna.kaczmarczyk@awf.edu.pl (K.K.); 2 Department of Sports Theory, Jerzy Kukuczka Academy of Physical Education, 40-065 Katowice, Poland; langfort@imdik.pan.pl; 3 Department of Kinesiology, Institute of Sport, 01-982 Warsaw, Poland; 4 Center for Sports Cardiology, Gajda-Med Medical Center in Pultusk, 06-100 Pultusk, Poland; gajda@gajdamed.pl; 5 Department of Kinesiology and Health Prevention, Jan Dlugosz University in Czestochowa, 42-200 Czestochowa, Poland

**Keywords:** hypoxia, testosterone, cortisol, anabolic–catabolic response, altitude training

## Abstract

The aim of this study was to analyze the effects of the “live high, train low” method (LH–TL) and intermittent hypoxic training (IHT) on testosterone (T) and cortisol (C) levels in cyclists. Thirty cyclists participated in the experiment. The LH–TL group (*n* = 10) was exposed to normobaric hypoxia (FiO_2_ = 16.3%) for 11–12 h a day and trained in normoxia for 3 weeks. In the IHT group (*n* = 10), participants followed the IHT routine three times a week for 3 weeks in normobaric hypoxia (FiO_2_ = 16.3%). The control group (N; *n* = 10) followed the same training protocol in normoxia. The LH–TL training was found to significantly increase (*p* < 0.05) T levels and the testosterone/cortisol (T/C) ratio during the experiment. The area under the curve (AUC) calculated for T levels over 4 weeks was significantly (*p* < 0.05) higher in the LH–TL group, by 25.6%, compared to the N group. The results also indicated a significant correlation (r = 0.53; *p* < 0.05) between AUC for T levels over 4 weeks and ∆ values of hemoglobin (HGB) in the LH–TL group. Overall, the findings show that LH–TL training at a moderate simulated altitude contributes to an increase in T levels and T/C ratio in athletes, which is a beneficial change stimulating anabolic processes and erythropoiesis.

## 1. Introduction

Endogenous hormones play an important role in the regulation of cellular metabolism during exercise and post-exercise recovery and are essential for inducing acute or chronic adaptive responses induced by physical exercise [1,2]. Testosterone (T) and cortisol (C) are important hormones that regulate anabolic and catabolic processes in the human body. Testosterone and cortisol are also key biomarkers of metabolic balance and indicators of current muscle status; monitoring their levels allows for the selection of training loads and recovery methods to optimize athletes’ exercise capacity [3].

Testosterone, an anabolic hormone, is essential for promoting protein synthesis and reducing protein breakdown, increases the ability of muscles to replenish glycogen stores, and contributes to increased red blood cell production. Cortisol, which acts antagonistically to testosterone, inhibits protein synthesis, increases protein degradation, and exhibits immunosuppressive effects [1,2,3,4]. Since testosterone and cortisol have opposing effects, it has been proposed that the T/C ratio can be used to assess the anabolic–catabolic balance in the body [5].

Both acute bouts of exercise and regular training can modify the activity of the autonomic nervous system and the hormonal profile. The extent of these changes is primarily determined by the type of training, training loads, and the sports level of athletes [6,7,8,9]. However, the findings of previous research on the anabolic–catabolic response to endurance training remain inconclusive. Some studies conducted on endurance athletes reported a training-induced decrease in resting T levels. This decrease can occur after just a few days, as a result of significant increases in both training intensity and training volume [10,11] or following long-term regular endurance training [12,13]. However, some studies have failed to support a decrease in T levels as a response to endurance training [6,14,15,16]. Previous findings on resting C levels are also inconclusive. Several studies [13,16] have indicated that endurance training may increase C levels or not cause significant changes in values of this hormone [10,14,15,17].

Altitude/hypoxic training has been used for many years to improve the performance and exercise capacity of athletes in various sports [18,19,20,21,22,23,24]. The methodology has been continually modified in search of greater effectiveness. As a result, three different concepts of high-altitude training have been adopted: “live high, train high” (LH–TH), “live high, train low” (LH–TL), and intermittent hypoxic training (IHT). These methods differ primarily in the time of hypoxic exposure, the conditions in which the training takes place (in hypoxia, or normoxia), and the type of hypoxia (hypobaric or normobaric). The LH–TH procedure assumes that the athlete lives and trains at altitude. In the LH–TL method, the athlete is exposed to hypoxia for 8–14 h/day (mainly while sleeping), whereas training is performed in normoxia. During IHT, in turn, the athlete resides in normoxia but performs training in hypoxic conditions [18,19,20,21,22,23,24].

For years, almost the only environment used by athletes and recommended by coaches to improve physical fitness was training under hypobaric hypoxia (high altitude training). In recent years, however, in order to avoid certain problems related to logistics, safety and invasive blood sampling collection, training under normobaric hypoxia has been applied for well-trained athletes. In general, both environmental conditions induce similar physiological changes in response to similar training process. This applies to the hemoglobin concentration (HGB), blood oxygen saturation (SpO_2_), oxygen consumption (VO_2_), and alveolar–arterial PO_2_ difference [25]. However, the only relevant condition that differentiates these two settings is the partial pressure of oxygen (PO_2_). Many researchers believe that this fact may be the reason for a different response to the same effort performed under normobaric hypoxia compared to hypobaric hypoxia. The best-documented differences are minute ventilation and blood NO metabolites [25].

One of the goals of altitude training is to increase the blood’s oxygen-carrying capacity in order to improve sea-level endurance performance in athletes. The elevated erythropoietin (EPO) production in hypoxia is a key factor enabling the subsequent improvement of hematological variables. The rate of EPO increase and acceleration of erythropoiesis depend on the duration of exposure and degree of hypoxia. The hematological response to altitude training is also affected by other factors, including the anabolic–catabolic balance, training loads, and iron store and supplementation [26]. However, knowledge regarding the anabolic–catabolic balance during and after training in hypoxia is still scarce. Although an increase in both T and C levels is observed immediately after acute exercise in hypoxia, findings indicate that a single exercise session performed in hypoxia may result in higher physiological stress and a more pronounced increase in C compared to exercise performed in normoxia [27,28,29]. However, the direction of changes in T and C induced by several weeks of altitude training remains unclear. Previous studies have mainly focused on changes in T and C levels following exposure to terrestrial altitudes above 3500 m [30,31,32,33]. Furthermore, a few studies have analyzed the effects of moderate altitude training on resting T and C levels, and their results are contradictory [34,35,36,37,38,39,40,41].

The anabolic–catabolic response of the athlete’s body to hypoxia may be one of the factors that determine the effectiveness of altitude training [26]. However, there is a lack of well-controlled studies dealing with the influence of recognized altitude/hypoxic training methods on changes of T and C levels. Therefore, the main novelty of this study was to investigate the effect of hypoxic training methods on the anabolic–catabolic response in well-trained athletes under strict control—with the training schedule, sleeping time, diet, nutrition supplements, and time of blood collection all being controlled in this study.

We adopted the hypothesis that the combination of hypoxia and physical exercise may contribute to beneficial changes in anabolic–catabolic response in athletes. Therefore, the aim of this study was to investigate the effect of the “live high, train low” (LH–TL) and intermittent hypoxic training (IHT) methods on blood T and C levels. For further insight into the anabolic–catabolic state of the athlete’s body, the value of the T/C ratio was also estimated.

## 2. Materials and Methods

### 2.1. Study Participants

Thirty well-trained male cyclists were recruited for this study. All athletes had current medical examinations, without any contraindications to performing exhaustive exercise in a hypoxic environment. The participants provided their written, voluntary, and informed consent before participation. Study participants were randomized into three groups: two experimental groups and a control group. The first experimental group (LH–TL) was exposed to normobaric hypoxia (FiO_2_ = 16.5%, ~2000 m) at rest and during sleep for 11 to 12 h a day for 4 weeks. Training in this group was performed in normoxia (200–300 m ASL). In the second experimental group (IHT), participants followed an IHT routine three times a week for 4 weeks in normobaric hypoxia (FiO_2_ = 16.5%, ~2000 m). The control group (N) lived and trained under normoxic conditions (200–300 m ASL). Thirty athletes met all the criteria and completed the whole experiment (Table 1).

The research project was conducted according to the Helsinki Declaration and was approved (No. 10/2015; approval date: 6 November 2015) by the Ethics Committee for Scientific Research at the Jerzy Kukuczka Academy of Physical Education in Katowice, Poland.

### 2.2. Experimental Design and Training Program

Throughout the experiment, all athletes followed the same training schedule, sleeping time, and diet. The participants consumed a controlled mixed diet (50% CHO, 20% fat, and 30% protein). Daily energy intake was set at ~3500 kcal. Athletes did not take any nutrition supplements.

During the experiment, the participants performed a 4-week training program. The LH–TL group was exposed to normobaric hypoxia in the evening and during sleep (11–12 h/day), using a hypoxic chamber. The fraction of inspired oxygen (FiO_2_) was 16.3%, corresponding to an altitude of ~2000 m. Training in this group was performed only under normoxic conditions. The IHT group lived in normoxia and performed training in normobaric hypoxia (FiO_2_ = 16.3%) 3 times a week in a hypoxic chamber. The control group (N), in turn, both lived and trained in normoxia.

The experiment was performed at the end of a preparation period, following a one-week recovery microcycle. The training program included three microcycles (3 weeks) with progressive training loads and a one-week recovery microcycle before the last series of tests. All the groups followed the same training routines, with individually adjusted intensity zones (Table 2). Intensity during these sessions was adjusted individually to each study participant based on the lactate threshold workload (WR_LT_) determined under normoxia (LH–TL, IHT, N groups), or under hypoxia for IHT training in the laboratory (IHT group). The WR_LT_ in normoxia and hypoxia was determined by Dmax method [42]. Our previous studies [43,44] demonstrated that LT determined by using the D-max method corresponds to the maximal lactate steady state (MLSS).

Each training session in the laboratory (T1, T2, and T3; three times per week; LH–TL and N group—normoxia, FiO_2_ = 20.9%; IHT group—hypoxia, FiO_2_ = 16.3%) included a 15 min warm-up, a 30-to-40 min main part, and a 15 min cool-down. Intensity during these sessions was adjusted individually to each study participant based on the LT workload determined under normoxia (WR_LT_; LH–TL, N groups) or under hypoxia (WR_LThyp_; IHT group). The warm-up during all laboratory sessions was performed at an intensity level of 65–70% WR_LT_/WR_LThyp_. In the main part, the intensity was increased to 100% WR_LT_/WR_LThyp_. This intensity level was maintained for 30 min (first week), 35 min (second week), and 40 min (third week). The cool-down included 15 min of continuous exercise at an intensity of 65–70% WR_LT_/WR_LThyp_. After completion of the final part of the laboratory session, cyclists performed a two-hour ride under normoxic conditions at an intensity level of 60–75% WR_LT_.

Other training sessions included low-intensity endurance exercise and resistance exercise in normoxia in all groups (Table 2). The recovery microcycle in all groups was conducted in normoxia. Training load before and during the experiment was recorded by using power meters (PowerTap, CycleOps, Madison, WI, USA). The training load was calculated after each training session and expressed on the Training Stress Score (TSS) point scale, using WKO + 3.0 software (TrainingPeaks, Louisville, CO, USA). During the experiment, all groups had their venous blood collected several times (see below for details).

The training program for the LH–TL and IHT groups during the experiment was based on our previous studies [18,23,24,38,45], which demonstrated a beneficial effect of the LH–TL and IHT protocols on aerobic capacity and sports performance.

### 2.3. Measurements during the Experiment

During the experiment, venous blood was drawn several times (baseline measurement; after the 1st, 2nd, and 3rd week of the experiment; and after the recovery week) from all participants. Each time, 10 mL of venous blood was collected from the antecubital vein under fasting conditions between 7:00 and 7:30 am. The hematological markers (red blood cell count, RBC; hemoglobin level, HGB; hematocrit, HCT; and blood reticulocyte percentage, Ret) were determined by using an Advia 2120 analyzer (Siemens, Erlanger, Germany). The blood collection after weeks 1, 2, and 3 and the recovery week was always performed after an active rest day. Creatine kinase (CK) activity and uric acid levels (URIC) were determined immediately after blood collection (Piccolo Express Chemistry Analyzer, Abaxis, Union City, CA, USA). The remaining blood samples were used to obtain serum after being left at room temperature for 0.5 h. The serum was frozen and stored at −70 °C until analyses. Serum levels of T and C were determined by radioimmunoassay, using Testosterone RIA and Cortisol RIA kits (Beckman Coulter).

Before blood baseline measurement, study participants performed a graded exercise test (40 W/3 min), using the Excalibur Sport cycle ergometer (Lode, Groningen, The Netherlands), in order to measure VO_2max_ (MetaLyzer 3B-2R, Cortex, Leipzig, Germany) and WR_LT_. These data were used to determine an individual training workload for the experiments. Furthermore, after 48 h of rest, the IHT group performed the same exercise test on the cycle ergometer under normobaric hypoxia conditions in order to determine the individual training load for the IHT workouts (WR_LThyp_). Measurement-taking during the experiment, as well as the graded exercise test protocol, was the same as reported in our previous studies [23,24,45].

During all test series and all training sessions in the laboratory (T1, T2, and T3), the atmospheric conditions in regard to temperature (19–19.5 °C), humidity (45–50%), concentration of carbon dioxide (450–600 ppm), and concentration of oxygen (FiO_2_ = 16.3%—IHT group) were controlled and held constant to increase the reliability of the investigations. The time of day and the order of participants were also recorded and remained the same for all participants in all series of testing.

### 2.4. Statistical Analysis

The results of the experiment were analyzed by using Statistica 13.0 software (TIBCO Software Inc., Palo Alto, CA, USA). The results are presented as arithmetic means (x) with standard deviations (SD). The Lilliefors test was used to demonstrate the consistency of the results with normal distribution. Significant differences in mean values between the initial values of the study groups were assessed by a one-way analysis of variance (ANOVA). The intergroup differences between consecutive research series were determined by using a two-way ANOVA (group and training) with repeated measures. The significance of differences between individual research series (differences between weeks) in the study groups was calculated by using Tukey’s post hoc test. The area under the curve (AUC) for T levels over 4 weeks in the LH–TL group was calculated by using the trapezoid method. Significant differences for AUC values between groups were determined by using a one-way ANOVA. Significance of differences between the study groups was calculated based on Tukey’s post hoc test. The paired samples t-test was used to determine the significance of differences in selected hematological variables in the LH–TL group. The relationships between AUC for T levels and changes in selected hematological variables in the LH–TL group were analyzed by using Pearson’s correlation coefficient. Statistical significance was set at *p* < 0.05. The a priori analysis (GPower 3.1 software,) [46] showed that, for *n* = 30, while maintaining an acceptable power (1 − β = 0.80) and α = 0.05, the ANOVA with repeated measures allows for detection of the effect size > 0.23.

## 3. Results

### 3.1. Changes in Testosterone and Cortisol Levels

**Figure 1:**
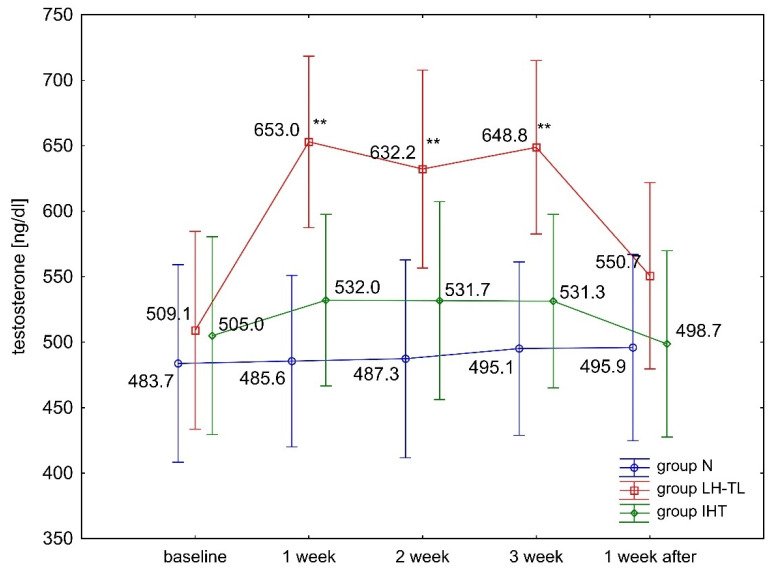
Blood serum testosterone levels (T) in experimental (LH–TL and IHT) and control (N) groups during the first experiment; ** *p* < 0.01—statistically significant differences compared to baseline.

**Figure 2:**
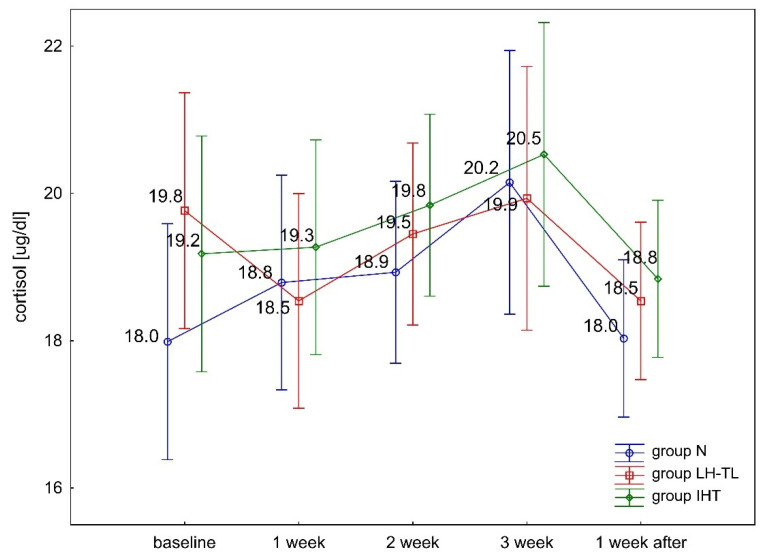
Blood serum cortisol levels (C) in experimental (LH–TL and IHT) and control (N1) groups during the first experiment.

**Figure 3:**
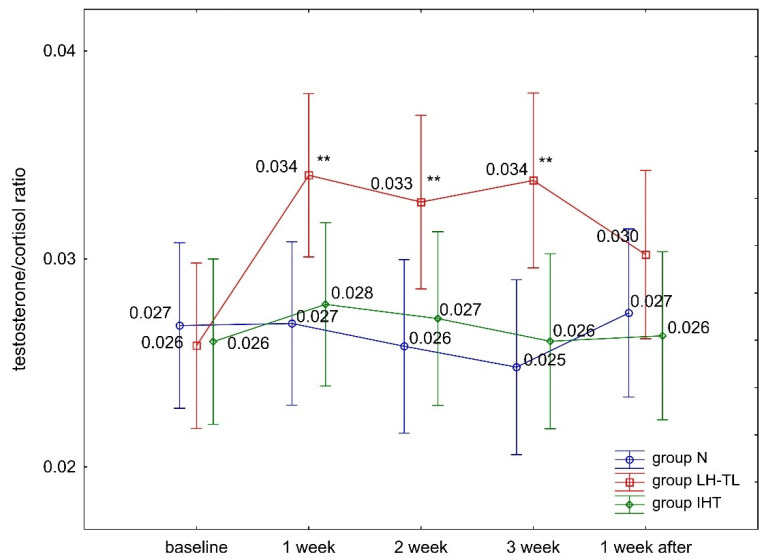
Blood serum testosterone/cortisol ratio (T/C) in experimental (LH–TL and IHT) and control (N) groups during the experiment; ** *p* < 0.01—statistically significant differences compared to baseline.

The ANOVA with repeated measures for group × training interactions showed statistically significant differences in blood serum T levels (F = 7.663, *p* < 0.001) and the (T/C) ratio (F = 3.638, *p* < 0.001). Tukey’s post hoc test revealed a significant (*p* < 0.01) increase in serum T levels in the LH–TL group. The levels of this hormone were significantly (*p* < 0.01) higher compared to baseline (from 24.1% to 28.2%) during regular exposure to hypoxia (Figure 1). During the recovery microcycle (performed in normoxia), serum T concentrations returned to baseline levels. The other study groups (IHT, N) showed no statistically significant differences in T levels during the experiment (Figure 1). There were no statistically significant differences in blood serum C levels in any of the study groups (IHT, LH–TL, and N) (Figure 2). No change in C levels resulted in a significant (*p* < 0.01) increase in the T/C ratio in the LH–TL group (Figure 3). A significant (*p* < 0.01) increase in the T/C ratio in LH–TL was observed after the first, second, and third training week (with 25.9%, 22.2% and 25.9%, respectively) (Figure 3). The other study groups (IHT and N) showed no statistically significant changes in the T/C ratio.

**Figure 4:**
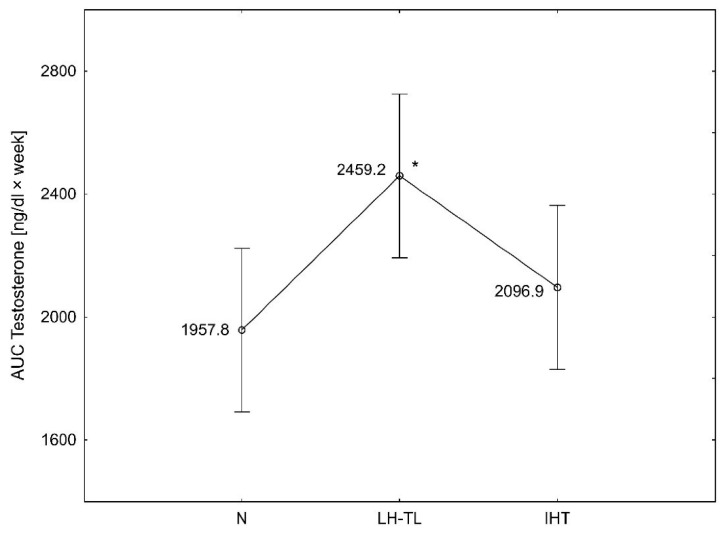
Area under the curve (AUC) calculated from testosterone levels over 4 weeks in experimental (LH–TL and IHT) and control (N) groups; * *p* < 0.05—statistically significant differences.

When time-course changes in serum T levels over 4 weeks were compared by using AUCs, the AUC values were significantly different between the three groups (F = 3.973, *p* < 0.05). The AUC values for testosterone levels over 4 weeks were significantly (*p* < 0.05) higher in the LH–TL group compared to N group by 25.6% (Figure 4).

### 3.2. Changes in Training Load and Biochemical Variables

The ANOVA showed no statistically significant group × training interactions for changes in CK activity and URIC levels during the experiment. However, the ANOVA revealed a statistically significant effect of training on changes in CK activity (F = 18.375, *p* < 0.001) and URIC levels (F = 21.465, *p* < 0.001) in the study groups. The statistical analysis showed no significant differences in training load (TSS) in the study groups (Table 3).

Tukey’s post hoc test showed a significant (*p* < 0.05) increase in blood serum CK activity after the second and third weeks of training in the study groups (IHT, LH–TL, and N). Furthermore, a significant (*p* < 0.05) increase in CK activity was found after the first week in the N group. Despite the significant increase in the activity of this enzyme, its levels were within the normal range in all groups. Similar changes were also observed in URIC levels in the study groups (Gr-IHT, Gr-LH–TL, and N). Statistically significant (*p* < 0.05) changes in URIC levels were observed after the second and the third training week. After the recovery microcycle, the CK activity and URIC concentration returned to baseline.

### 3.3. Changes in Selected Hematological Variables in LH–TL Group

The analysis of selected hematological variables demonstrated significant (*p* < 0.001) improvements in the red blood cell count (RBC), hemoglobin concentration (HGB), hematocrit (HCT), and blood reticulocyte percentage (Ret) in the LH–TL group. During the last series of examinations (1 week after exposure to hypoxia), the RBC was found to increase by 6.3%, HGB by 6.6%, HCT by 4.5%, and Ret by 38% compared to baseline values. Changes in the above indices were not observed in the IHT and N groups (Table 4).

**Figure 5:**
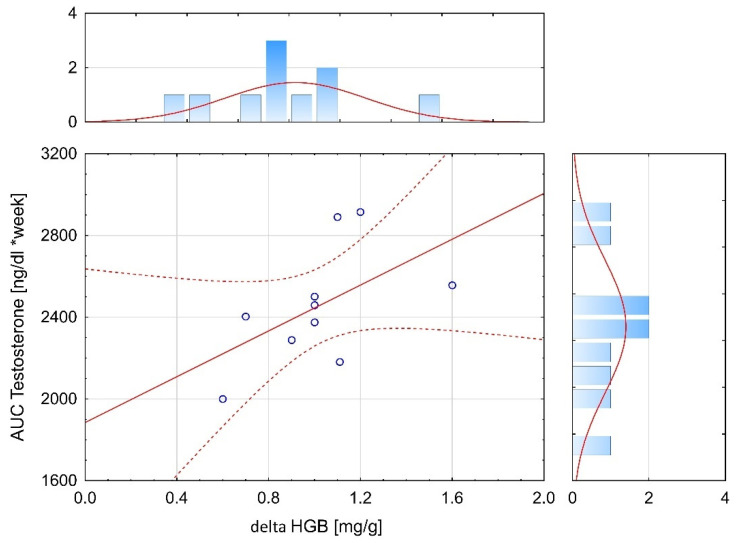
Correlation between area under the curve (AUC) for testosterone levels over 4 weeks (AUC testosterone) and delta values of hemoglobin concentration (∆HGB) during the experiment in the LH–TL group.

The results indicated a significant correlation (r = 0.53, *p* < 0.05) between AUC for T levels over 4 weeks and ∆ values of HGB in the LH–TL group (Figure 5). No significant correlations were found between the AUC for T levels over 4 weeks and the ∆ values of RBC, HCT, and Ret in the LH–TL group.

## 4. Discussion

The main contribution of the present study is to provide new and relevant data regarding the effects of LH–TL and IHT training methods carried out in normobaric hypoxia on T and C levels in endurance athletes. Overall, our findings indicate that training at a moderate simulated altitude, using the LH–TL method, leads to elevated T levels and an elevated T/C ratio, indicating the increased stimulation of anabolic processes. The same phenomenon was not observed during IHT and normoxia training.

Altitude or hypoxic training has been incorporated into training programs in order to improve sea-level athletes’ sport performance or to ensure that athletes achieve better results in disciplines where competitions are held at altitude [23,45,47]. Although the reasons behind the efficacy of altitude/hypoxic training methods are still under debate, it is mostly, especially in the case of endurance training, attributed to favorable hypoxia-induced changes in erythropoietic response and hematological variables [26]. However, altitude/hypoxic training has been demonstrated to cause major physiological stress to the body, resulting in meaningful neuroendocrine system responses. The changes of plasma T and C levels and their ratio are postulated as relevant biomarkers for the anabolic–catabolic state of the body, indicating the training status of the athletes [5]. However, the response of T and C after training performed in hypoxic conditions still remains poorly understood.

### 4.1. Testosterone

Previous studies on the effects of the hypoxic environment on T levels have primarily focused on changes in T levels induced by staying at high altitudes. Basu et al. [31] observed a decrease in T levels in elite climbers after ascent from 3500 to 5000 m and after long-term residence (7 weeks) at altitudes >5200 m [32]. Similar reductions in blood T levels were noted following an 18-day stay at an altitude of 3500 m [48]. It has been suggested that negative energy balance may be an important factor responsible for reduced blood T levels at high altitudes [49]. Indeed, it was shown that, following 21 days of exposure to an altitude of 4300 m, blood T levels increased and remained elevated only in the group with a balanced and controlled diet. In contrast, in groups remaining on negative energy balance residing in both hypoxia and normoxia, T levels decreased significantly during the experiment [50].

Data on changes in T levels following altitude training at moderate altitudes are scarce and inconclusive. An increase in resting T levels was observed in speed skaters following 2 weeks of training at an altitude of 2000 m [35]. In contrast, it was found that blood T levels in cyclists decreased after 31 days of “live high, train high” (LH–TH) training at 2690 m [39]. However, no changes in T levels were found in elite cross-country skiers and biathletes after 14–18 days of training at an altitude of 1650 m [36]. Furthermore, a study by Humpeler et al. [50] on hikers showed an increase in blood T levels 48 h after arrival at 2000 m, whereas on days 5 and 10 of the stay, and on return to the lowlands, T levels were no longer significantly different from baseline.

In our study, a significant increase in blood T levels, was observed during training by the LH–TL method. This increase in T levels occurred after the first week of training and then remained at elevated levels for the 3 weeks of the experiment. Similar changes were not found in the IHT and N group. One week after completion of altitude training, however, T levels were not different than baseline values; this result is consistent with the results reported by Vasankari et al. [36] and Humpeler et al. [50]. Although Vasankari et al. [36] reported no changes in T levels after returning from high altitude, they noted that resting T levels recorded during camp (on day 8 or 15) were significantly higher than baseline levels; this is also in agreement with the results of our study.

It is likely that the decrease in T levels observed by Gore et al. [39] as a response to altitude training was due to the excessive training load used in the LH–TH protocol. This is also reflected by the occurrence of illnesses and injuries in all participants during or after their stay at altitude. Based on this observation, it is worth noting that the direction of changes in T levels may depend on the altitude-training protocol used. The LH–TH protocol, which involves a constant stay in hypoxia, may cause a decrease [39,51] or inhibit an increase in T levels [50], possibly as a result of the excessive stress on the athlete’s body. The LH–TL method used in our study involves a shorter exposure to hypoxia per day, leading to an increase in T levels and maintaining high T levels throughout training camp. However, it seems that there is a minimum threshold time of exposure to hypoxia below which an increase in T levels does not occur. This conclusion is based on the fact that, during our experiment, IHT training, where the exposure time to hypoxia was short (~9 h; 1 h/day, 3 × per week), did not cause significant changes in T levels.

Higher T levels not only increase protein synthesis and reduce protein breakdown but may also contribute to a higher rate and extent of hematological adaptive changes induced by altitude training. Indeed, it is believed that T may stimulate the hematopoietic system by increasing erythropoietin synthesis and secretion, acting on erythroid cells in the bone marrow, improving iron absorption and transport, stimulating iron incorporation into erythrocytes, increasing hemoglobin synthesis, prolonging erythrocyte survival time, and increasing 2,3-diphosphoglycerate (2,3-DPG) levels in red blood cells [52,53,54]. Our results showed a positive correlation (r = 0.53; *p* < 0.05) between AUC for T levels over 4 weeks and ∆ values of HGB in LH–TL group. Based on our results, we believe that the increase in T levels observed in our study during altitude training is a change that may further stimulate an improvement in the blood’s oxygen-carrying capacity, contributing to an increase in athletes’ performance and exercise capacity. Nevertheless, no significant correlations were found between AUC for T levels and ∆ values of RBC, HCT, and Ret in the LH–TL group. This indicates that the importance of changes in T levels in the improvement of hematological variables following altitude training should be the subject of further research.

### 4.2. Cortisol

It is widely accepted that both acute and sustained hypoxia stimulate the adrenal cortex, leading to an increase in the plasma level of C [36,37]; however, a few studies have failed to report a change in plasma cortisol [38], especially in normobaric hypoxia [55]. This unresolved issue was addressed in early studies on altitude training [36,37] and led to the conclusion that training using LH–TH and LH–TL methods performed under terrestrial moderate altitudes contributed to an increase in blood C levels in elite athletes. On the other hand, Wilber et al. [40] suggest that the gradual increase in C levels seen over 6 weeks of the LH–TH protocol was primarily due to the progressively increasing training load rather than due to hypoxia exposure alone. This conclusion was based on insignificant changes in C concentration seen after the first 24 to 36 h at altitude and is also partially in line with results obtained after 7 h of exposure to moderate altitude (normobaric hypoxia; FiO_2_ = 15.0%) that showed no changes in C levels and no difference from those recorded in normoxia [56].

In our study, neither of the altitude/hypoxic training protocols employed (neither LH–TL nor IHT) caused statistically significant changes in blood C levels in the athletes after each training microcycle. However, it should be emphasized that, to minimize the effect of training load on changes in blood C levels, measurements in subsequent microcycles were performed after a day of active rest each time. The suggestion made by Wilber et al. [40] that changes in C levels may be due to an increase in training load rather than hypoxia alone was confirmed in our study by a similar tendency for changes in C levels in the control group (N) as compared to those observed in groups subjected to altitude training (LH–TL and IHT). It could be speculated that the reduction in oxygen availability during exercise in hypoxia results in a higher accumulation of C, due to a greater participation of anaerobic metabolism. Under normal physiological conditions, a large accumulation of C occurs during high-intensity anaerobic exercise, which is often associated with damage to the cells of working muscles. The latter phenomenon usually results in elevated blood level of CK, commonly recognized as related to greater skeletal muscle damage. Because changes in blood CK were similar in both experimental conditions, our results additionally support the view of Wilber et al. [40] that normobaric hypoxia has no significant effect on C secretion during the training process under hypoxic conditions. This conclusion is additionally supported by the similar changes in blood URIC level in both experimental groups. The level of blood URIC is known to depend on the intensity of the exercise performed: the greater the intensity of exercise, the higher the blood URIC level. In sum, the increase in C levels was accompanied each time by a similar increase in blood CK and URIC levels in response to applied training.

Our results are in agreement with several previous studies that involved several weeks of training at moderate altitude. Tiollier et al. [42] demonstrated that, in highly trained cross-country skiers, the LH–TL procedure (3 × 6 days with a simulated altitude of 2500, 3000, and 3500 m; 11 h/day) did not change resting C levels. Changes in C levels were also not observed after the LH–TH procedure in cross-country skiers and biathletes residing and training for 18–28 days at an altitude of 1600–1800 m [38] or in elite cross-country skiers after 14 days at 2000 m [34].

These previous findings, together with the results of the present study, suggest that moderate normobaric hypoxia does not significantly increase blood C levels. However, this seems not to be the case for subjects exposed to high altitudes (above 3500 m) [29,57], thus possibly indicating that the severity of hypoxic stimulus plays an important role in changes in C levels.

### 4.3. T/C Ratio

It has been recommended that the T/C ratio should be used to assess the anabolic–catabolic balance in the athlete’s body [5]. The T/C ratio is considered to be an indicator that is more sensitive to training load than T and C levels separately, whereas chronic decreases in the T/C ratio may reflect increased protein degradation or inhibition of protein synthesis [3]. High C levels and a significant decrease in the T/C ratio during altitude training may also contribute to a decrease in the rate of erythropoiesis in the bone marrow, especially during the early acclimation phase [58].

In our study, we observed that altitude training in the LH–TL group resulted in an increase in the T/C ratio that was due to an increase in T levels and non-significant changes in C levels. This change indicates the occurrence of a hormonal anabolic milieu in the blood of athletes that was not observed in the normoxic group. Feng et al. [51] reported that, despite a different direction of hormonal changes than in our experiment, an increase in the T/C ratio was also observed in elite runners during and after 4 weeks of training at an altitude of 1917 m. An increase in the T/C ratio was also observed in ice skaters after training at 2000 m [35].

Our study provides evidence that altitude training based on LH–TL methods performed at a moderate simulated altitude can favorably change the T/C ratio and stimulate anabolic processes in endurance athletes. However, in view of the results presented in this paper, properly selected training loads seem to be the decisive factor here.

## 5. Limitations

There are two major limitations to this study, and they could be addressed in future research. Firstly, our study focused on the assessment of the anabolic–catabolic balance in exposure to normobaric hypoxia. This raises the question of whether different physiological responses may be possible between hypobaric and normobaric hypoxia. Opinions are still divided on this issue [59,60,61]. Saugy et al. [62] indicated that the post-LH–TL hematological responses and exercise performance improvements were similar for hypobaric and normobaric stimuli. It has also been reported that exercise in the normobaric and hypobaric hypoxic environments produced a similar pattern of changes in cortisol and growth hormone levels [29,63]. Nevertheless, several studies [25] have reported a number of variables (e.g., minute ventilation and blood NO metabolites) that were different between the normobaric and hypobaric hypoxic conditions, lending support to the notion that true physiological differences are indeed present.

However, the small number of previous studies comparing hypobaric and normobaric hypoxia does not allow us to conclude clearly whether the type of hypoxia significantly affects the difference in anabolic–catabolic balance during altitude training. Secondly, in our study, the hypoxic level corresponded to an altitude of 2000 m. It is of interest whether the T/C ratio would increase after exposure to altitudes above 2000 m.

In the present study, training schedule, sleeping time, diet, nutrition supplements, and time of blood collection were under strict control. The blood collection was always performed after a day off, under fasting conditions, between 7:00 and 7:30 am, when cortisol and testosterone levels are the highest during the day. This procedure minimized the influence of the circadian rhythm, meals consumed, and training loads on the results. However, our results do not allow us to conclude whether the T/C ratio might also increase in different phases of the circadian rhythm.

Further randomized and crossover studies are recommended, seeking a deeper understanding of the effects of such interventions.

## 6. Conclusions

The results of our study indicate that LH–TL training at moderate simulated altitudes contributes to an increase in T levels and T/C ratio in athletes; these are beneficial changes leading to the stimulation of anabolic processes and an increase in the rate of erythropoiesis. However, to maintain the beneficial direction of anabolic–catabolic changes, it is necessary to select a training load adjusted to the current training level of athletes, a proper altitude training method, and an appropriate level of hypoxia.

The most prominent practical implication of these findings is that T and C levels, as well as the T/C ratio, should be monitored during altitude/hypoxic training, so as to ensure that adaptive changes occur effectively.

## Figures and Tables

**Table 1:** Characteristics of the study participants (mean ± SD).

	LH–TL *n* = 10	IHT *n* = 10	N *n* = 10	Results of One-Way ANOVA
Age (y)	20.5 ± 2.9	20.7 ± 3.1	21.8 ± 4.0	F = 0.230 *p* = 0.977
Height (cm)	181 ± 4.3	178.0 ± 5.3	178.2 ± 3.4	F = 1.750 *p* = 0.193
Weight (kg)	69.6 ± 3.9	67.5 ± 5.3	68.1 ± 4.8	F = 0.442 *p* = 0.647
FAT (%)	8.4 ± 2.6	10.6 ± 2.0	8.4 ± 2.4	F = 2.903 *p* = 0.082
VO_2max_ (mL/kg/min)	66.0 ± 4.1	67.6 ± 2.7	67.0 ± 2.9	F = 0.541 *p* = 0.844
WR_LT_ (W)	292 ± 21.4	286.0 ± 25.0	280 ± 21.1	F = 2.154 *p* = 0.255

Abbreviations: FAT—body fat content; VO_2max_—maximal oxygen consumption; WR_LT_—workload at lactate threshold.

**Table 2:** Training program during the experiment.

Day	Microcycle 1	Microcycle 2	Microcycle 3	Microcycle 4
1	T1 + 2 h endurance training (60–75% of WR_LT_)	T2 + 2 h endurance training (60–75% of WR_LT_)	T3 + 2 h endurance training (60–75% of WR_LT_)	Day off
2	3–4 h of endurance training 60–75% of WR_LT_ with high-speed intervals (2 × 6 × 10 s-max)	3–4 h of endurance training 60–75% of WR_LT_ with high-speed intervals (2 × 6 × 10 s-max)	3–4 h of endurance training 60–75% of WR_LT_ with high-speed intervals (2 × 6 × 10 s-max)	1 h active recovery ride < 55% WRLT
3	T1 + 2 h endurance training (60–75% of WR_LT_)	T2 + 2 h endurance training (60–75% of WR_LT_)	T3 + 2 h endurance training (60–75% of WR_LT_)	2 h of endurance training 60–75% of WR_LT_ with high-speed intervals (2 × 6 × 10 s-max)
4	Strength endurance (gym) Upper body	Strength endurance (gym) Upper body	Strength endurance (gym) Upper body	Strength endurance (gym) Upper body
5	T1 + 2 h endurance training (60–75% of WR_LT_)	T2 + 2 h endurance training (60–75% of WR_LT_)	T3 + 2 h endurance training (60–75% of WR_LT_)	T1 + 1 h endurance training (60–75% of WR_LT_)
6	3–4 h of endurance training 60–75% of WRLT with high-speed intervals (2 × 6 × 10 s-max)	3–4 h of endurance training 60–75% of WRLT with high-speed intervals (2 × 6 × 10 s-max)	3–4 h of endurance training 60–75% of WRLT with high-speed intervals (2 × 6 × 10 s-max)	2 h of endurance training 60–75% of WRLT
7	Day off	Day off	Day off	Day off

Abbreviations: T1—training in the laboratory (15 min of warm-up (65–70% WR_LT_/WR_LThyp_ ), 100% WR_LT_/WR_LThyp_ for 30 min and 15 min of cool-down at 65–75% WR_LT_/WR_LThyp_; T2—training in the laboratory (15 min of warm-up (65–70% WR_LT_/WR_LThyp_ ), 100% WR_LT_/WR_LThyp_ for 35 min and 15 min of cool-down at 65–70% WR_LT_/WR_LThyp_; T3—training in the laboratory (15 min of warm-up (65–70% WR_LT_/WR_LThyp_ ), 100% WR_LT_/WR_LThyp_ for 40 min and 15 min of cool-down at 65–70% WR_LT_/WR_LThyp_.

**Table 3:** Training load and changes in selected biochemical indices in the study groups (LH–TL, IHT, and N) during the first experiment; * *p* < 0.05—statistically significant differences compared to baseline.

Variables	Group	Measurement				
		Baseline (x ± SD)	1 Week (x ± SD)	2 Weeks (x ± SD)	3 Weeks (x ± SD)	4 Weeks (x ± SD)
Training load (TSS)	LH–TL	462 ± 35	1094 * ± 63	1147 * ± 73	1283 * ± 69	412 ± 27
	IHT	451 ± 25	1128 * ± 49	1164 * ± 69	1276 * ± 76	387 ± 22
	N	434 ± 29	1152 * ± 51	1194 * ± 76	1308 * ± 86	426 ± 31
CK (U/I)	LH–TL	90.1 ± 34.3	139.3 ± 48.9	159.7 * ± 58.3	161.7 * ± 62.1	98.7 ± 27.7
	IHT	110.9 ± 32.1	151.8 ± 52.6	168.1 * ± 64.1	175.1 * ± 52.7	115.8 ± 34.1
	N	89.3 ± 28.4	147.2 * ± 42.6	158.8* ± 49.4	161.7 * ± 55.7	108.4 ± 32.2
URIC (mg/dL)	LH–TL	4.75 ± 0.34	4.98 ± 0.24	5.65 * ± 0.26	5.81 * ± 0.31	4.79 ± 0.34
	IHT	4.83 ± 0.29	5.12 ± 0.34	5.74 * ± 0.38	5.97 * ± 0.42	4.89 ± 0.29
	N	4.91 ± 0.38	5.21 ± 0.41	5.84 * ± 0.46	6.01 * ± 0.52	4.87 ± 0.31

Abbreviations: CK—creatine kinase, URIC—uric acid.

**Table 4:** Selected hematological variables in the experimental LH–TL group during initial and final evaluations; * *p* < 0.05, ** *p* < 0.01—statistically significant differences compared to the baseline values.

Variable	Baseline (x ± SD)	After 4 Weeks (x ± SD)	∆ (x ± SD)
RBC (million/μL)	5.01 ± 0.2	5.33 ** ± 0.23	0.32 ± 0.19
HGB (g/dL)	15.3 ± 0.67	16.3 ** ± 0.76	1.0 ± 0.27
HCT (%)	44.5 ± 2.3	46.5 ** ± 2.5	2.0 ± 1.08
Ret (%)	1.00 ± 0.19	1.38 * ± 0.13	0.38 ± 0.2

Abbreviations: RBC—red blood cell count; HGB—hemoglobin concentration; HCT—hematocrit; Ret—blood reticulocyte percentage.

## Data Availability

The data presented in this study are available on request from the corresponding author.
